# Ardipusilloside-I Metabolites from Human Intestinal Bacteria and Their Antitumor Activity

**DOI:** 10.3390/molecules201119719

**Published:** 2015-11-19

**Authors:** Wei-Yu Cao, Ya-Nan Wang, Peng-Yuan Wang, Wan Lei, Bin Feng, Xiao-Juan Wang

**Affiliations:** 1 State Key Laboratory of Military Stomatology, Department of Pharmacy, School of Stomatology, The Fourth Military Medical University, Xi’an 710032, China; weiyu_cao@163.com (W.-Y.C.); peng_yuanwang@163.com (P.-Y.W.); wanlei_fmmu@163.com (W.L.); binfengfmmu@163.com (B.F.); 2 State Key Laboratory of Bioactive Substance and Function of Natural Medicines, Institute of Materia Medica, Chinese Academy of Medical Sciences and Peking Union Medical College, Beijing 100050, China; wangyanan@imm.ac.cn

**Keywords:** ardipusilloside-I, human intestinal bacteria, metabolism, UHPLC–MS, HPLC–NMR, antitumor activity

## Abstract

Ardipusilloside-I (ADS-I) is a triterpenoid saponin extracted from *Ardisia pusilla* DC, and has been demonstrated to have potent antitumor activity. However, ADS-I metabolism in humans has not been investigated. In this study, we studied the biotransformation of ADS-I in human intestinal bacteria, and examined the *in vitro* antitumor activity of the major metabolites*.* Ultra-high performance liquid chromatography–tandem mass spectrometry (UHPLC–MS/MS) was used to detect ADS-I biotransformation products, and their chemical structures were identified by high performance liquid chromatography–nuclear magnetic resonance (HPLC–NMR). The antitumor activity of the major metabolites was determined by the MTT assay. Here, we show that main reaction seen in the metabolism of ADS-I in human intestinal bacteria was deglycosylation, which produced a total of four metabolites. The structures of the two major metabolites M1 and M2 were confirmed by using NMR. MTT assay showed that ADS-I metabolites M1 and M2 have the same levels of inhibitory activities as ADS-I in cultured SMMC-7721 cells and MCF-7 cells. In conclusion, this study demonstrates deglycosylation as a primary pathway of ADS-I metabolism in human intestinal bacteria, and suggests that the pharmacological activity of ADS-I may be mediated, at least in part, by its metabolites.

## 1. Introduction

**Figure 1:**
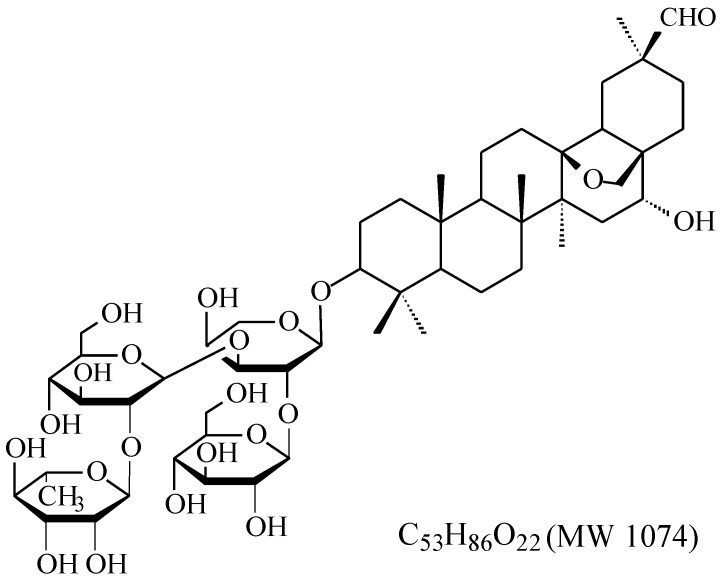
Chemical structures of ADS-I.

Ardipusilloside-I (ADS-I, Jiujielong in Chinese) (Figure 1) [1] is a triterpenoid saponin isolated from *Ardisia pusilla* DC (*Mysinaceae*). It has been demonstrated that ADS-I has potent inhibitory activity against the growth of many types of tumor, such as the liver, stomach, ovarian, and lung tumors [2]. Recently, our group has shown that ADS-I significantly suppresses the growth of rat glioma (C6) both *in vitro* and *in vivo* [3]. However, just like most other saponins, ADS-I may cause hemolysis, which makes it unsuitable for disease treatment through intravenous administration. Animal studies indicate that ADS-I has significant inhibitory effects on mouse sarcoma (S37, S180), Lewis lung cancer and liver cancer in nude mice (SMMC-7721) after oral administration [4,5,6,7], but in our previous study, like most other natural saponins, ADS-I had a poor intestinal absorption and was barely absorbed through the gastrointestinal tract after oral administration in rats [8], which limits the development of this compound as a new drug candidate. In these studies four possible deglycosylated metabolites were found in the contents from rat small intestine [8]. A number of studies have demonstrated that the bioactive substances derived from natural saponins are mostly their metabolites produced by intestinal bacteria [9,10,11,12]. These findings imply that the metabolites of ADS-I may be the key components for its inhibitory activity against the growth of tumor in animal experiments after oral administration. Thus, an understanding of saponin metabolites that have potent antitumor activity as well as favorable pharmacokinetic properties could be a new strategy for developing effective antitumor agents.

Although the metabolism of ADS-I in rats was investigated, the metabolites of ADS-I in human intestine have not reported, which however is required as part of the preclinical development of oral anticancer drugs based on ADS-I. In this study, we investigated the products of ADS-I biotransformation by human intestinal bacteria *in vitro* through extraction, separation, and purification. Subsequently, we analyzed and deduced the structures of those metabolites by high performance liquid chromatography–evaporative light scattering detector (HPLC–ELSD), ultra high performance liquid chromatography–electrospray ionization–tandem mass spectrometry (UHPLC–ESI–MS/MS) and high performance liquid chromatography–nuclear magnetic resonance (HPLC–NMR) technology, intending to clarify the possible ADS-I metabolism pathways in human intestinal bacteria. Furthermore, we compared the anti-tumor activities of ADS-I with its metabolites in both cultured human liver carcinoma cell line SMMC-7721 and breast carcinoma cell line MCF-7.

## 2. Results

### 2.1. HPLC–ELSD Analysis

**Figure 2:**
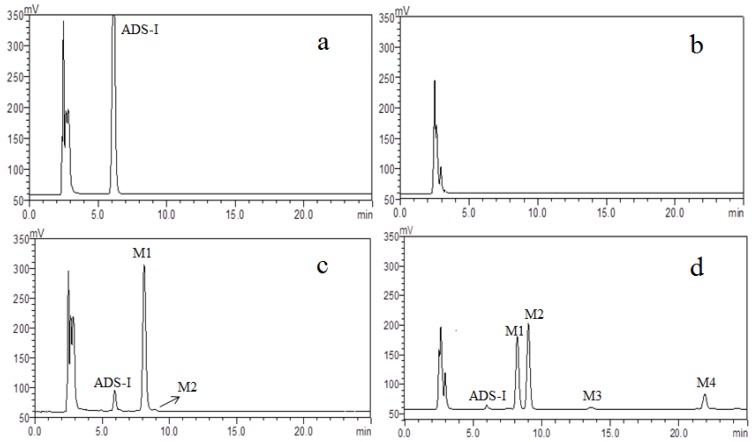
HPLC–ELSD chromatograms of (**a**) 0 h control incubation with ADS-I; (**b**) 72 h control incubation without ADS-I; (**c**) 24 h incubation with ADS-I; (**d**) 72 h incubation with ADS-I.

Several metabolites of ADS-I were found in cultures of human intestinal bacteria, indicated by the decrease of the ADS-I peak with the time of incubation, and several unknown peaks were observed following incubation from 0 to 72 h as compared to ADS-I control. The strong peak appearing at an early retention time was the peak from the ingredients of the general anaerobic medium. As shown in Figure 2, the main metabolites of ADS-I were M1 at 24 h and then M1 was gradually transformed to metabolites M2 and M3, M4 in 72 h. These data suggested that under the anaerobic culture conditions, ADS-I was transformed into new compounds by human intestinal bacteria metabolism. According to the retention time, we inferred that the polarity of these metabolites are less than that of ADS-I. This result coincides with the previous studies, showing that triterpenoid saponins were converted to secondary glucosides or aglycones through deglycosylation and they have less polar properties in human intestinal bacteria biotransformation [13,14,15,16]. The metabolites of ADS-I were used for subsequent structural determination by UHPLC–MS, HPLC–NMR analysis.

### 2.2. UHPLC–MS Analysis

According to the literature reports, triple quadrupole (QQQ) mass spectrometers can provide relatively abundant fragment ions by appropriately adjusting the fragmentor voltage [17,18,19]. In this study, we used ESI–MS and set a high fragmentor voltage (F = 380 V) to analyse the human intestinal bacteria metabolites of ADS-I in both positive and negative ionization modes. The results showed that the negative ion mode provided higher sensitivity, thus we used negative ion mode in subsequent experiments. According to the above HPLC–ELSD chromatogram results (Figure 2), we found that larger amounts of metabolites were harvested at 72 h compared with other incubation times. Thus we selected the metabolism of 72 h samples for MS analysis by UHPLC–MS. 

**Figure 3:**
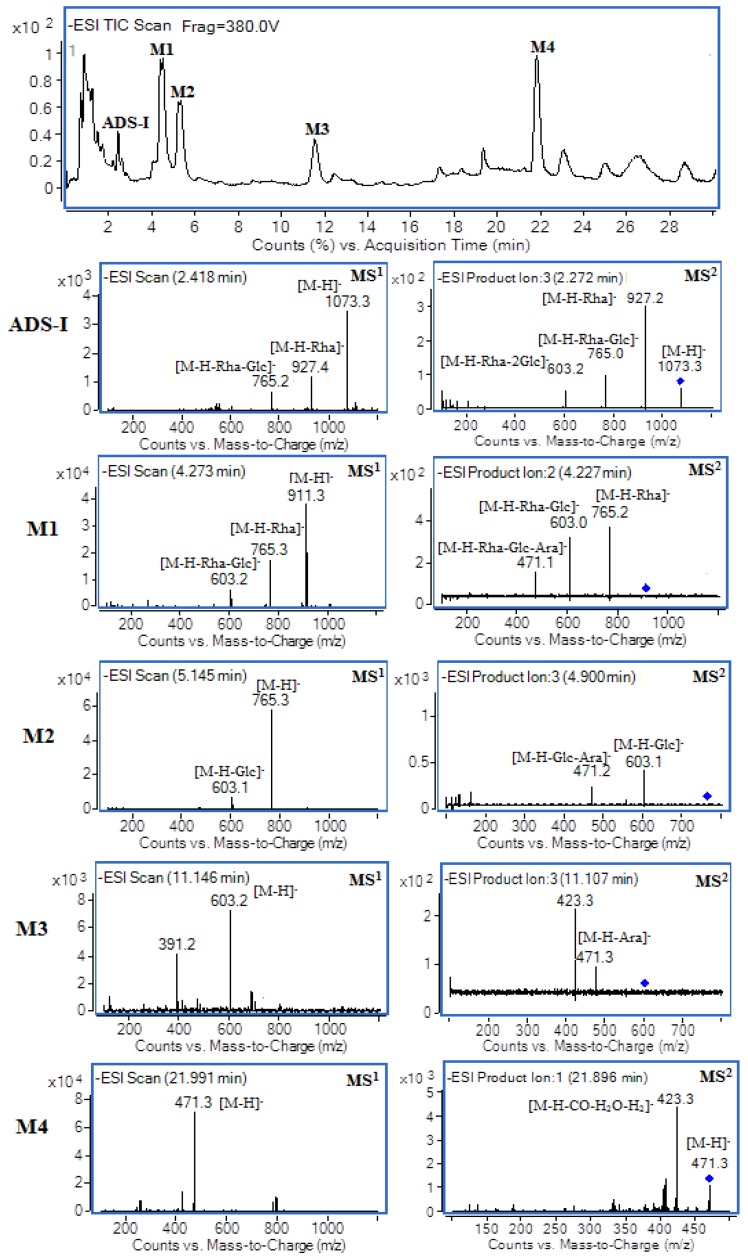
The typical total ion chromatogram and mass spectra (MS^1^ and MS^2^) of ADS-I and its metabolites formed by human intestinal bacteria.

This experiment showed the typical deprotonated molecular ions [M − H]^−^ (*m/z* 1073, 911, 765, 603, 471) of ADS-I and its metabolites M1–M4 by full–UHPLC–ESI–MS (Figure 3). More characteristic fragment ion information about ADS-I and its metabolites could be gained through adjusting the mass spectral fragmentor voltage and collision energy (40 V~58 V). This indicated that the deprotonated molecular ions [M − H]^−^ *m/z* 1073 of the ADS-I could produce fragment ions *m/z* 927, 765, 603 and the precursor ion [M – H − Rha]^−^ *m/z* 927 could produce ions *m/z* 765,603,471 by MS^2^. The mass spectrum (MS^1^) of M1 showed the deprotonated molecular ions [M − H]^−^ at *m/z* 911,which was 162 Da less than that of ADS-I (*m/z* 1073) corresponding to the loss of one glucose molecule. Its MS^2^ data showed ions at *m/z* 765 ([M − H − Rha]^−^), *m/z* 603 ([M − H − Rha − Glc]^−^) and *m/z* 471 ([M − H − Rha − Glc − Ara]^−^), thus it could be inferred that M1 was an ADS-I deglycosylated metabolite formed by loss of the terminal glucose. The metabolite M2 ion [M − H]^−^ *m/z* 765, which was 146 Da less than M1, produced the ions *m/z* 603 ([M − H − Glc]^−^), *m/z* 471 ([M − H − Glc − Ara]^−^). According to the HPLC–ELSD results, we concluded that M2 might be a transformed product of M1 and it could be inferred that M2 was transformed from M1 by losing one rhamnose. Similarly, it could be concluded that M3 was transformed from M2 by losing a glucose and M4 was transformed from M3 by further losing one arabinose. Based on the chemical structure of the ADS-I and the fragment ion information obtained by multistage mass spectrometry, we inferred the structure of the metabolites, and preliminarily concluded that M1–M4 were deglycosylated ADS-I metabolites. The collected retention time, molecular weight and multistage mass spectrometry information data are shown in Table 1.

**Table 1:** The chromatography and mass spectrometry data of ADS-I and its metabolites.

ADS-I and Its Metabolites	*TR* (min)	Possible Formula	Molecular Mass	[M − H]^−^ (*m*/*z*)	MS^2^ Fragments
ADS-I	2.4	C_53_H_86_O_22_	1074	1073	[1073]: 927, 765, 603
					[927]: 765, 603, 471
M1	4.3	C_47_H_76_O_17_	912	911	[911]: 765, 603, 471
					[765]: 603, 471
M2	5.1	C_41_H_66_O_13_	766	765	[765]: 603, 471
M3	11.1	C_35_H_56_O_8_	604	603	[603]: 471, 407
M4	22.0	C_30_H_48_O_4_	472	471	[471]: 423, 405, 377
					[423]: 405, 375

### 2.3. Sephadex LH-20 Chromatography

The metabolic samples of ADS-I in human intestinal bacteria were harvested after incubation for 72 h, and were purified by using a Sephadex LH-20 column with MeOH–H_2_O (1:1, *v*/*v*) as mobile phase at a flow rate of 0.5 mL/min. After separation, each fraction was analyzed by HPLC–ELSD and UHPLC–MS, and the same fractions were pooled, and further purified by chromatography on a C_18_ column again.

### 2.4. HPLC–SPE–NMR Analysis

**Figure 4:**
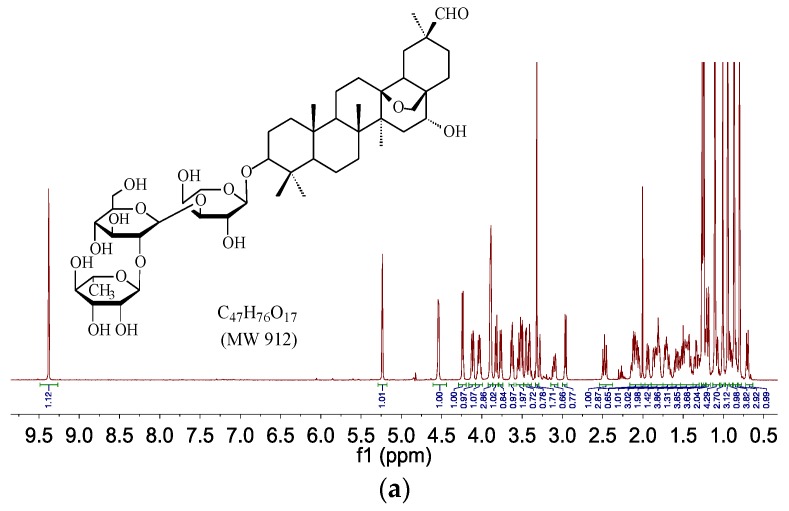

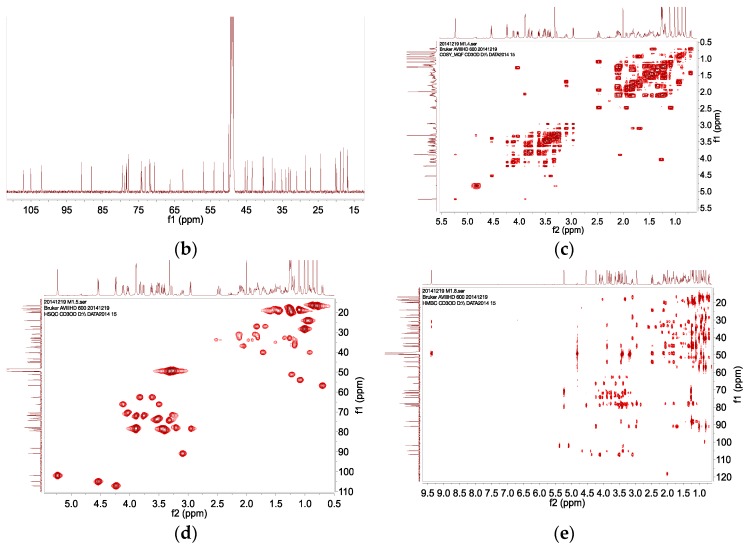
^1^H-NMR (**a**); ^13^C-NMR (**b**); ^1^H-COSY (**c**); HSQC (**d**); and HMBC (**e**) of ADS-I intestinal bacteria metabolite M1.

The ^1^H-NMR and ^13^C-NMR spectra of M1 demonstrated that it was a glycoside derivative of cyclamiretin A. In the ^1^H-NMR spectrum (CD_3_OD, 600 MHz, Figure 4a), δ_H_ 5.23 (d, *J* = 1.9 Hz, 1H), 4.54 (d, *J* = 7.1 Hz, 1H), 4.24 (d, *J* = 6.5 Hz, 1H), 1.26 (d, *J* = 6.2 Hz, 3H) and δ_H_ 2.94–4.15 showed the presence of an α-rhamnose, a β-glucopyranose, and an α-arabinose, further demonstrated by ^13^C-NMR (Figure 4b), HSQC (Figure 4d) and HMBC (Figure 4e) spectra. The signals at δ_H_ 1.26 (d, *J* = 6.2 Hz, 3H) were assigned to a CH_3_ of the α-rhamnose. The ^13^C-NMR (CD_3_OD, 150 MHz) displayed 47 carbon resonances, or six carbon atoms less compared with ADS-I. ^13^C-NMR signals (δ_C_ 107.1, 105.0 and 102.0) confirmed the presence of the α-rhamnose, a β-glucopyranose, and α-arabinose. Compared with ADS-I (molecular mass at 1074), the mass spectrum showed that the molecular weight of M1 was 912, indicating it was formed by losing a glucose from ADS-I. Based on the above data and comprehensive 2D-COSY (Figure 4c) experiments, the structure of M1 was elucidated as the glycoside derivative of cyclamiretin A formed by the loss of a glucose from ADS-I, and was identified as 3-*O*-[α-*L*-rhamnose (1→2)-β-d-glucopyranose (1→3)]-α-*L*-arabinose–cyclamiretin A (Figure 4).

**Figure 5:**
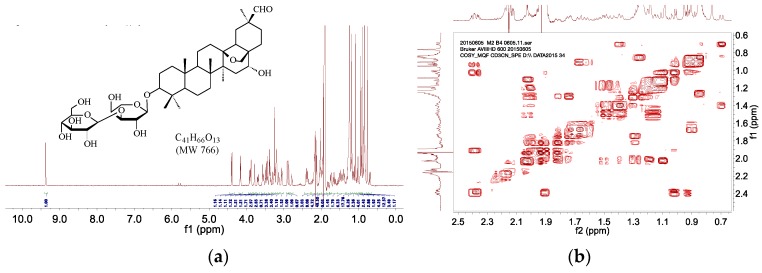

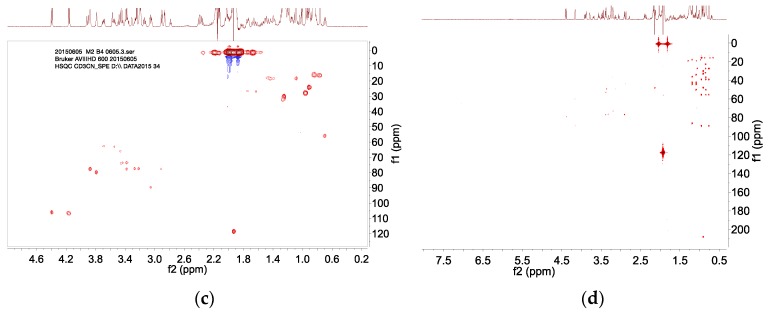
^1^H-NMR (**a**); ^1^H-COSY (**b**); HSQC (**c**); and HMBC (**d**) of ADS-I intestinal bacteria metabolite M2.

The NMR spectra of M2 displayed that M2, M1 and ADS-I were analogues (glycoside derivatives of cyclamiretin A). The ^1^H-NMR spectrum (CD_3_CN, 600 MHz, Figure 5a) of M2 showed signals corresponding to a β-glucopyranose and an α-arabinose (δ_H_ 4.39, 1H, d, *J* = 7.8 Hz; 4.16, 1H, d, *J* = 7.2 Hz). Furthermore, the signals at δ_C_ 106.4 and 105.9 in the HSQC spectrum (Figure 5c) were consistent with the presence of β-glucopyranose and α-arabinose, respectively. Compared with M1, the ^1^H-NMR spectrum of M2 lacked the signals of the anomeric proton at δ_H_ 5.23 (d, *J* = 1.9 Hz, 1H) and the methyl proton at 1.26 (d, *J* = 6.2 Hz, 3H) from rhamnose, which indicated that M2 was a glycoside derivative of cyclamiretin A formed by the loss of a rhamnose from M1. Comparing with M1 (molecular mass at 912), the molecular weight of M2 was 766, which was deduced to result from the loss of a rhamnose from M1. On the basis of 2D-COSY (Figure 5b) HMBC (Figure 5d) and MS experiments, the structure of M1 was inferred to correspond to a glycoside derivative of cyclamiretin A consisting of two sugars formed by losing a rhamnose from M1. Therefore, M2 was confirmed as 3-*O*-[β-d-glucopyranose (1→3)]-α-*L*-arabinose-cyclamiretin A (Figure 5).

### 2.5. Cytotoxicity Assay

**Figure 6:**
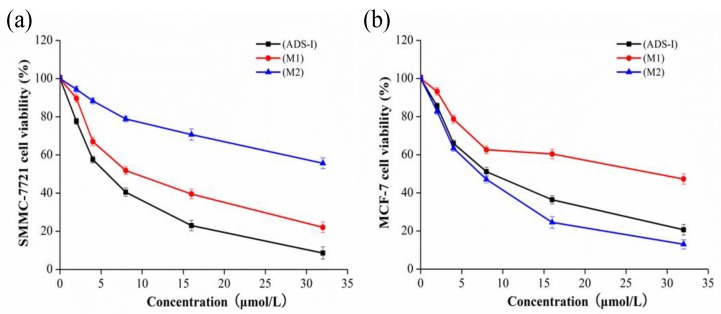
Effect of ADS-I and its metabolites (M1, M2) on the cell viability of human carcinoma cells. (**a**) Viability of the human hepatocellular carcinoma cell line SMMC-7721 cells treated by different concentrations of ADS-I and its metabolites (M1, M2) for 72 h; (**b**) Viability of the human breast carcinoma cell line MCF-7 cells treated by different concentrations of ADS-I and its metabolites (M1, M2) for 72 h. Values are expressed as means ± SD (*n* = 3).

The effect of ADS-I and its metabolites (M1, M2) on the viability of human hepatocellular carcinoma cell line SMMC-7721 cells and breast carcinoma cell line MCF-7 cells were assessed by the MTT assay. As shown in Figure 6, ADS-I and its metabolites (M1, M2) showed dose-dependent inhibition on SMMC-7721 and MCF-7 cell growth for 72 h. The IC_50_ values of ADS-I, M1, M2 on SMMC-7721 cells were 5.48, 9.65, 40.91 μmol/L, respectively, so we inferred that the activity of ADS-I was gradually decreased by human intestinal bacteria metabolism, but the metabolite M1 still had a significant activity in the inhibition SMMC-7721 cell growth. The IC_50_ values of ADS-I, M1, M2 on MCF-7 cells were 8.77, 24.10, 6.76 μmol/L, respectively. We thus found that the cytotoxic activity of M2 is higher than that of ADS-I in MCF-7 cells.

## 3. Discussion

**Figure 7:**
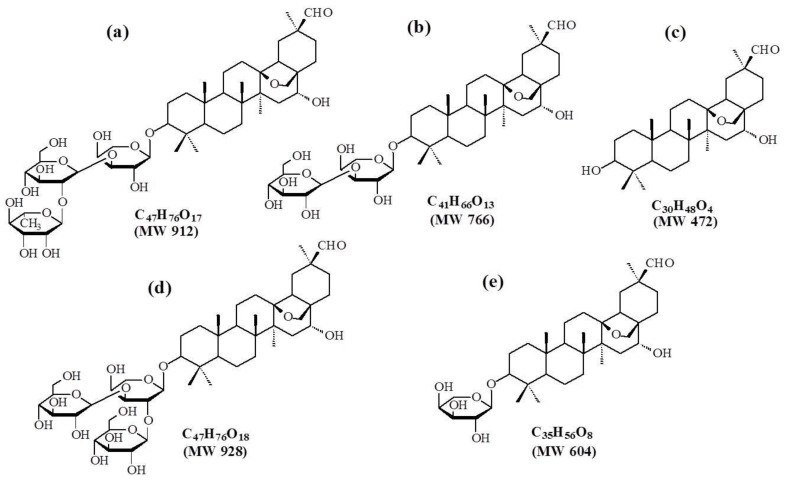
The possible structures of ADS-I intestinal bacteria metabolites, (**a**–**c**) the same metabolites of ADS-I by human and rat intestinal bacteria respectively; (**d**) the unique metabolite of ADS-I by rat intestinal bacteria; (**e**) the unique metabolite of ADS-I by human intestinal bacteria.

In this study, we investigated the *in vitro* biotransformation of ADS-I by human intestinal bacteria. A total of four metabolites were detected by UHPLC–QQQ–MS, and the two most abundant metabolites were separated and purified by a Sephadex LH-20 chromatography column. We identified the chemical structures of the two novel metabolites by HPLC–NMR. The results show that the main metabolic pathway of ADS-I by human intestinal bacteria is deglycosylation through stepwise cleavage of sugar moieties. Compared with the metabolites of ADS-I produced by rat intestinal bacteria reported in reference [8], the metabolites of ADS-I by human intestinal bacteria are slightly different. There are three identical metabolites of ADS-I produced by both human intestinal bacteria and rat intestinal bacteria which are the secondary glycosides and aglycone obtained through deglycosylation reactions by cleavage of the end of glucose moieties (Figure 7a), the (terminal glucose + rhamnose) moieties (Figure 7b) and the (terminal glucose + rhamonse + glucose + arabinose) moieties (Figure 7c) from ADS-I, respectively. However, there is a unique rat intestinal bacteria metabolite of ADS-I produced though loss of rhamonse moieties (Figure 7d) and a unique human intestinal bacteria metabolite of ADS-I produced by losing the (terminal glucose + rhamnose + glucose) moieties (Figure 7e). The possible chemical structures of ADS-I intestinal bacteria metabolites are given in Figure 7. It indicated that the biotransformation of ADS-I by different intestinal bacteria are not exactly same because of the differences between human and animal intestinal microflora composition, as different species of intestinal bacteria can produce different metabolites by biotransformation of ADS-I, due to their different enzyme systems.

Furthermore, we have separated and purified two novel metabolites M1, M2 of ADS-I by human intestinal bacteria through Sephadex LH-20 chromatography. We studied the antitumor activity of ADS-I and its metabolites M1, M2 on the human hepatocellular carcinoma cell line SMMC-7721 cells and the breast carcinoma cell line MCF-7 cells by MTT assays. The study shows that the metabolites M1, M2 all have inhibitory activity on SMMC-7721 cells and MCF-7 cells, M1 has stronger activity on SMMC-7721 cells while M2 has stronger activity than ADS-I on MCF-7 cells. The explanation of this different cytotoxicity of these two metabolites is not simple. Our data show that M2 is a transformed form of M1 produced by losing one rhamnose, indicating that they have a different structure. Even though the structures are slightly different, they may target different molecules or the same molecule(s) but with different affinities. Also, different tumor cells (SMMC-7721 *vs.* MCF-7) may develop different molecular pathways for their survival and growth, resulting in different responses to these compounds. A similar finding was reported in the literature. The ginseng saponin Rb1 metabolic pathway was Rb_1_→Rd→F_2_→Compound K by human intestinal bacteria, and F_2_ and Compound K only differ in one glucose moiety, but Compound K has stronger anti-tumor activities both *in vivo* and *in vitro* [20,21]. In addition, the animal studies indicate that ADS-I has significant inhibitory effects, on Lewis lung cancer and liver cancer in nude mice (SMMC-7721) after oral administration [3,4,5,6,7], but in our previous study, ADS-I was barely absorbed through the gastrointestinal tract after oral administration in rats [8]. We inferred that the metabolites of ADS-I produced by intestinal bacteria might play important roles in the activities of ADS-I after oral administration, and the intestinal absorption and bioavailability of the metabolites could be more favorable than that of ADS-I, so the results provide a reference for understanding the biotransformation and absorption of ADS-I in the human intestine for further clinic research.

In view of the above points, we conclude that ADS-I metabolites have a more significant inhibitory effect than ADS-I in several tumor-bearing animal experiments after oral administration of ADS-I. This work may help interpret pharmacokinetic data of ADS-I, and provide scientific evidence for the mechanism of ADS-I absorption in the human intestinal system. The work also provides the theoretical basis for further study of the antitumor activity of ADS-I metabolites *in vivo* in clinical research.

## 4. Experimental Section

### 4.1. Chemicals and Reagents

ADS-I (purity > 95%) [22] was provided by the Department of Pharmacy, School of Stomatology, Fourth Military Medical University (Xi’an, China). General anaerobic medium (GAM) was purchased from Qingdao Hope Bio-Technology (Qingdao, China). HPLC grade acetonitrile and methanol were from Fisher Scientific (Pittsburgh, PA, USA). Deionized water (18.2 MΩ) was supplied with analytic ultra-pure water system (ELGA, High Wycombe, UK). SephadexLH-20 and methylthiazolyl tetrazolium salt (MTT) were from Solarbio (Beijing, China). Dulbecco’s Modified Eagle’s medium (DMEM) and fetal bovine serum (FBS) were from HyClone Laboratories (Logan, UT, USA), and Penicillin-streptomycin and 0.25% trypsin-EDTA solutions were from Solarbio (Beijing, China). Other reagents were of analytical purity.

### 4.2. Preparation of Human Intestinal Bacterial Specimen

The GAM was prepared for fermentation experiments. In brief, GAM medium (9.6 g) was dissolved in deionized water and then the pH was adjusted to 7.2 with 0.5 M NaOH solution, and it was adjusted to a total volume of 1000 mL. The resultant anaerobic medium was autoclaved at 121 °C for 20 min. Human fecal specimens were prepared according to previous methods [23,24], fresh human fecal sample was obtained from a healthy female volunteer who was not on any medication for three months, and had not drank alcohol or smoked for 48 h before fecal collection. A 10 g fecal sample was weighed and mixed with 50 mL of anaerobic dilution medium. After being homogenized and then centrifuged at 500× *g* for 5 min, the supernatant was centrifuged at 10,000× *g* for 30 min. The resulting precipitates were mixed with 10 mL anaerobic culture medium as the human intestinal bacteria fraction.

### 4.3. Metabolism of ADS-I by Human Intestinal Bacteria

The biotransformation of ADS-I by human intestinal bacteria was determined in a 50 mL incubation system containing 1 mL human intestinal bacteria culture solution and 10 mg ADS-I powder (dissolved in 200 μL methanol) in anaerobic dilution medium. Incubation without ADS-I human intestinal bacteria served as blank control. All samples in the incubation system were anaerobically incubated at 37 °C for 0, 24, 48, 72 h, respectively. Reactions were stopped by adding 50 mL water-saturated *n*-butyl alcohol–ethyl acetate (1:1) mixture, and extraction was performed three times. Supernatant layers were combined and evaporated under reduced pressure at 75 °C. The residues were dissolved in methanol and centrifuged at 10,000× *g* for 10 min. The supernatant was analyzed by HPLC–ELSD and UHPLC–MS.

### 4.4. HPLC–ELSD Analysis

HPLC analysis of ADS-I metabolites were carried out using an LC-20A high performance liquid chromatograph (Shimadzu Corporation, Kyoto, Japan) equipped with a Alltech type 3300 evaporative light-scattering detector (Alltech Associates, Deerfield, MA, USA). A Diamonsil C_18_ (2) column (4.6 × 250 mm, 5 μm) from Diamonsil Technologies (Beijing, China) was used for all separations and the column temperature was maintained at 25 °C. The mobile phase consisted of (A) ultra-pure water and (B) methanol using a gradient elution of 75% B at 0–12 min, 75%–90% B at 12–30 min. The flow rate was 1 mL/min and the injection volume was 10 μL. The ELSD was set to a probe temperature of 60 °C, a gain of 1 and the nebulizer gas nitrogen at a flow of 2.0 L/min.

### 4.5. UHPLC–MS Analysis

UHPLC–MS analysis was performed using an Agilent 1290 Infinity ultra-high performance liquid chromatography (UHPLC) and 6460 type triple quadrupole (QQQ) mass spectrometer equipped with electrospray ionization source (ESI) and Mass Hunter working software (Agilent Technologies, Palo Alto, CA, USA). A Poroshell 120 EC C_18_ column (2.1 mm × 100 mm, 2.7 μm) from Agilent Technologies was used as an analytical column and the column temperature was maintained at 25 °C. The mobile phase consisted of (A) ultra-pure water and (B) acetonitrile using a gradient elution of 35% B at 0–15 min, 35%–50% B at 15–16 min, 50% B at 16–30 min. The flow rate was 0.4 mL/min and the injection volume was 2 μL. The ESI–QQQ–MS instrument was operated in the negative ion mode using an electrospray ionization source. The operating parameters were optimized as follows: drying gas (N_2_) flow rate, 10.0 L/min; drying gas temperature, 350 °C; nebulizer, 45 psi; capillary, 3500 V; fragmentor voltage, 380 V; sheath gas temperature, 350 °C; sheath gas flow rate, 11 L/min. Mass spectra were recorded across the range *m/z* 100–1200 in negative modes. The system was operated under Mass Hunter Acquisition Software version B.04.10 (Agilent Technologies).

### 4.6. The Separation and Purification of ADS-I Metabolites

ADS-I human intestinal bacteria metabolic samples were prepared using the same method as described above (Section 4.2 and Section 4.3). We selected the samples incubated for 72 h and applied them to a Sephadex LH-20 column (2.0 cm × 30 cm) eluted with MeOH–H_2_O (1:1, *v*/*v*) at a flow rate of 0.5 mL/min. The different effluents were collected (4 mL in each fraction). Similar fractions were combined according to the results of the HPLC–ELSD and UHPLC–MS. Finally, the pooled fractions were further purified by using a C_18_ chromatography column.

### 4.7. HPLC–SPE–NMR Analysis

HPLC–SPE–NMR measurements were carried out by using a chromatographic separation system consisting of an Agilent 1260 series HPLC with an in-line solvent degasser, quaternary pump, auto-sampler, column compartment with thermostat, and a diode array detector. NMR measurements were performed using a Bruker AVANCE III HD 600 MHz instrument. The chromatographic separation was carried out using a YMC-C_18_ column (4.6 mm × 250 mm, 5 μm), and the column temperature was maintained at 25 °C. The mobile phase consisted of 35% (A) ultra-pure water and 65% (B) methanol using a isocratic elution. The detection wavelength was 205 nm and 210 nm, and the flow rate was 1 mL/min. We obtained the high purity of metabolites M1 and M2 samples used the SPE device with on-line enrichment, and the structure of M1 and M2 were analyzed by NMR analysis.

### 4.8. Cell Culture

The human hepatocellular carcinoma cell line SMMC-7721 and breast carcinoma cell line MCF-7 were purchased from the Cell Resource Center of the Chinese Academy of Sciences Shanghai Institutes (Shanghai, China). SMMC-7721 and MCF-7 cells were cultured in DMEM medium supplemented with 10% FBS and 1% antibiotics (100 IU/mL penicillin and 100 μg/mL streptomycin). Cells were grown at 37 °C in a humidified 95% air and 5% CO_2_ atmosphere.

### 4.9. Cytotoxicity Assay

ADS-I and its metabolites M1, M2 were dissolved in dimethyl sulfoxide (DMSO) and stored at −20 °C, then thawed and diluted in DMEM prepared for treatment. In all experiments, the final DMSO concentration did not exceed 1‰ (*v*/*v*). The *in vitro* cytotoxicity was tested by MTT assay. In brief, cells were seeded in 96-well plates at a density of 5 × 10^3^ per well and were cultured at 37 °C for 24 h in a humidified 95% air and 5% CO_2_ atmosphere. After, ADS-I, M1, M2 (0, 2, 4, 8, 16, 32 μM) were added to each well, and cell cultures were grown for another 72 h. After further incubation with MTT (20 μL, 5 mg/mL) for 4 h, cells were dissolved in 150 μL DMSO per well, and the optical density (OD) was measured with an ELX800 reader (Bio-Tek instruments, Inc., Winooski, VT, USA) at 490 nm. Cells incubated without the test compounds were used as controls. The anti-proliferative activity was presented as the percent of reduction in cell viability, which was calculated by: Anti-proliferative activity = (OD_0_ − OD_x_)/OD_0_ × 100%, where OD_0_ represented the OD measurement of untreated cell cultures, and OD_x_ the OD of drug-treated cell cultures. The viability of the control cells from the untreated cultures was defined as 100% and the IC_50_ value was calculated by SPSS version 16.0.

### 4.10. Statistical Analysis

Data were expressed as means ± standard derivation (SD). Statistical analysis was performed using the statistical software SPSS 16.0 (SPSS Inc., Chicago, IL, USA). Student’s test was used to analyze statistical differences between groups. *p* < 0.05 was considered statistically significant. 
